# Incidence and Risk Factors for Retinal Detachment Following Cataract Surgery at a Tertiary Center in Jeddah, Saudi Arabia

**DOI:** 10.7759/cureus.33968

**Published:** 2023-01-19

**Authors:** Safaa I Beheiri, Abdullah S Alghorair, Fahad A Aljambi, Majed A Alotaibi, Mohammad A Alzahrani, Abdulaziz A Subyani, Majed M Alharbi, Abdulrahman B Aboalola, Terad A Alnajjar

**Affiliations:** 1 Department of Ophthalmology, King Abdulaziz University Hospital, Jeddah, SAU; 2 College of Medicine, King Abdulaziz University Hospital, Jeddah, SAU

**Keywords:** outcome, complications, risk factors, cataract surgery, retinal detachment

## Abstract

Introduction

The incidence of retinal detachment in the general population is 0.08%, it has been reported to reach 0.7% or higher following cataract surgery. One of the most important risk factors for retinal detachment is posterior capsular rupture during cataract surgery. Additional risk factors include high myopia, history of ocular trauma, young age, male sex, and diabetes. In this study, we aimed to investigate the incidence and risk factors of retinal detachment following cataract surgery in patients treated at our hospital.

Materials and methods

This retrospective cohort study was conducted at King Abdulaziz University Hospital (KAUH), a tertiary center in Jeddah, Saudi Arabia. The medical records of 365 patients (365 eyes) who underwent cataract surgery from 2017 to 2021 were reviewed.

Results

The average age of the 365 patients was 63 years, and 47.7% (n=174) of patients were male. The incidence of retinal detachment was 3.3% (n=12). The risk of RD was 2.8 times higher for the right eye than for the left eye. The incidence of RD was significantly more in eyes with intraoperative posterior capsular rupture, zonular dehiscence, corneal trauma, and surgery combined with anterior vitrectomy than in other eyes. The most common complication of cataract surgery is corneal edema, which was observed in 22.7% of the eyes; our analysis revealed a significant relationship between corneal edema and the duration of surgery.

Conclusion

In our study, we highlighted the higher incidence of retinal detachment compared to those in other studies; most cases occurred one month postoperatively.

## Introduction

Cataract surgery represents the most common surgical procedure in developed countries, as cataracts are a leading cause of blindness worldwide [1,2]. Retinal detachment (RD) is a moderately concerning complication following cataract surgery, as previous studies have reported that approximately half of the patients undergoing the procedure and having developed RD are associated with a final visual acuity no better than 20/40 [3]. The estimated risk of RD following cataract surgery is 0.7%, which is much higher than that for rhegmatogenous RD in the general population (0.08%) [4].

A previous retrospective study from Western Australia reviewed data collected from 129,982 patients who underwent cataract surgery over a period of 21 years. The overall RD rate after cataract surgery was 0.7%, consistent with previously published data [5]. Another four-year retrospective study among the general population in France reported an estimated RD incidence of 0.99% following cataract surgery, citing the type of cataract surgery as an important risk factor. Indeed, the authors reported a three-fold increase in the risk of RD in patients undergoing extracapsular extraction (3.87%) when compared with the risk after phacoemulsification (0.96%). Intraoperative posterior capsular ruptures were also among the most significant risk factors for RD (6.39%), along with high myopia (9.21%), history of eye trauma (7.58%), young age, male sex, and diabetes (1.21%) [6].

The period between the onset of RD and cataract surgery has varied among studies; a systematic review of 21 papers published from 1997-2017 demonstrated that the peak incidence mostly occurred from a few months to years postoperatively. Most included articles reported a mean interval between 12 and 40 months, although the largest study reported a range of 2.5-24 months [7]. Additional studies have reported a higher incidence of RD in the first six to 24 months after cataract surgery [8-10].

Given that RD after cataract surgery is uncommon, large datasets are required to obtain accurate estimates of its true incidence. The present study aimed to examine the incidence and risk factors of RD following cataract surgery in patients treated at King Abdulaziz University Hospital (KAUH) in Jeddah, Saudi Arabia.

## Materials and methods

This retrospective cohort study was conducted in the Department of Ophthalmology at King Abdulaziz University Hospital (KAUH), a tertiary center in Jeddah, Saudi Arabia. The study was approved by the Research Ethics Committee at KAUH. For this study, we reviewed the medical records of 365 eyes that underwent cataract surgery from 2017 to 2021, which were selected randomly out of 1002 eyes to have a 98% confidence level and a margin of error within 5%. Patients with untreated preoperative RD were excluded from the analysis.

The following data were collected from the medical records of each patient: age, nationality, operated eye, preoperative visual acuity, preoperative intraocular pressure, fundus exam results, type of cataract, past ocular history, past medical history, history of ocular surgery, type of cataract surgery, any combined procedure, intraoperative complications, duration of surgery, type of anesthesia, postoperative lens status (pseudophakic or aphakic), postoperative intraocular pressure, postoperative complications including retinal detachment, and postoperative visual acuity at the one-year follow-up.

Visual acuity data were converted to LogMAR values and categorized as follows based on the recommendations of the International Council of Ophthalmology (ICO): normal vision, 20/20 to 20/60 (LogMAR 0.1 to 0.5); moderate vision, 20/80 to 20/200 (0.6 to 1.0), and poor vision >20/200 (≥1.1).

SPSS version 21 (IBM Corp., Armonk, NY) was used for statistical analysis. Continuous variables are presented as the mean and standard deviation while categorical variables are presented as numbers and percentages. Student’s t-tests and correlation were used to evaluate the differences between continuous variables, and chi-square was used to evaluate categorical variables. A p-value of <0.05 was considered significant.

## Results

Baseline characteristics

The average age of the sample was 63 years, and 47.7% (n=174) of patients were male. Preoperatively, visual acuity was good, impaired, and poor in 47.3%, 35.5%, and 17.2% of cases, respectively, with a mean value of 0.6 (20/80). Fundus exam results were abnormal in 38.1% of patients, and the most commonly reported abnormality was diabetic retinopathy in 20.5% of patients (Table 1). The mean preoperative intraocular pressure was 15.4 mmHg (range: 4 to 40 mmHg). The most commonly operated type of cataract was nuclear sclerosis in 60% (n=219); other types and the mean duration of surgery are addressed in Table 2.

**Table 1:** Preoperative fundus abnormality

Number (percentage)	Abnormality
44 (20.5%)	Diabetic retinopathy
7 (3.3%)	Macular edema with diabetic retinopathy
3 (1.4%)	Macular edema
4 (1.9%)	Peripheral retinal changes
2 (0.9%)	Macular fibrosis
2 (0.9%)	Macular degeneration
1 (0.5%)	Macular hole
2 (0.9%)	Vascular abnormality
3 (1.4%)	Myopic fundus
3 (1.4%)	Extramacular drusen
7 (3.3%)	Disc cupping
2 (0.9%)	Pale disc
2 (0.9%)	Retinal detachment mark

**Table 2:** Cataract type and duration of surgery

Mean duration (min)	Number	Cataract type
42.07	219 (60%)	Nuclear cataract
38.32	22 (6%)	Posterior subcapsular cataract
40.25	4 (1.1%)	Cortical cataract
64.00	10 (2.7%)	Congenital cataract
43.18	11 (3%)	Mature cataract
31.50	2 (0.5%)	Hypermature cataract (morgagnian)
43.49	97 (26.6%)	Unspecified

Diabetes and hypertension were the most common comorbidities with rates of 58.1% (n=212) and 50.7% (n=185), respectively (Table 3). Analysis of ocular history indicated that 9.6% (n=35) of the eyes were glaucomatous, 6% (n=22) were myopic, 2.2% (n=8) were hyperopic, and 3.6% (n=13) had keratopathy. Only 5.2% (n=19) of patients had a previous surgical history for the same eye, and most cases involved posterior chamber surgeries (2.7%, n=10), including vitrectomy and retinal procedures. In addition, glaucoma surgery was performed in 1.1% of patients (n=4).

**Table 3:** Preoperative comorbidities

No	Yes	Comorbidities
153 (41.9%)	212 (58.1%)	Diabetes Mellitus
180 (49.3%)	185 (50.7%)	Hypertension
361 (98.9%)	4 (1.1%)	Vascular disease
298 (81.6%)	67 (18.4%)	Ischemic heart disease
329 (90.1%)	36 (9.9%)	Thyroid disease

Intraoperative information

The type of anesthesia was local in most of the patients (95.1%, n=347), although general anesthesia was used in 4.9% (n=18) of the patients. The average duration of surgery was 43 minutes, ranging from 11 to 145 minutes (Table 4). The longer surgical duration was significantly associated with lower postoperative visual acuity (Table 5).

**Table 4:** Surgical duration

Number (percentage)	Duration in minutes
25 (6.8%)	Less than 20 minutes
98 (26.8%)	20–30 minutes
88 (24.1%)	31–40 minutes
51 (14%)	41–50 minutes
41 (11.2%)	51–60 minutes
62 (17%)	More than 60 minutes

**Table 5:** Correlation between surgical duration and postoperative visual acuity PO: Postoperative

P value	R (strength)	
P=0.000	0.351	Duration of surgery & PO vision 1^st^ day
P=0.017	0.213	Duration of surgery & PO vision 1^st^ week
P=0.000	0.350	Duration of surgery & PO vision 1^st^ month
P=0.217	0.111	Duration of surgery & PO vision 1^st^ year

Anterior vitrectomy was combined with cataract surgery in 6.6% (n=24) of the eyes, posterior vitrectomy in 1.4% (n=5) of the eyes, and glaucoma surgery in 1.9% (n=7) of the eyes.

Secondary intraocular lenses were implanted in 98.1% of the eyes (n=358) while 1.9% (n=7) of the eyes were left as aphakic.

Intraoperative complications were reported in 6.8% (n=25) of the eyes, including posterior capsular rupture in 3% (n=11), zonular dehiscence in 2.5% (n=9), and vitreous loss in 0.5% (n=2) of the eyes. The remaining complications were vitreous hemorrhage, trauma to the iris, and trauma to the cornea in one eye each (0.3% each).

Postoperative results

Postoperatively, the incidence of RD was 3.3% (n=12), with 41.1% (n=5) of cases occurring after one month postoperatively, 33.3% (n=4) occurring after one year postoperatively, 16.6 % (n=2) occurring after one day postoperatively, and 8.3% (n=1) occurring after one week postoperatively, and we reported that the risk of RD was 2.8 times higher for the right eye than for the left eye. Postoperative visual acuity on Day 1 was good, impaired, and poor in 55.5%, 34.2%, and 10.3% of cases, respectively. After one year, postoperative visual acuity was good, impaired, and poor in 81%, 12.7%, and 6.3% of cases, respectively (Table 6). Also, we noticed that high preoperative IOP was significantly associated with lower postoperative visual acuity (P=0.032).

**Table 6:** Postoperative visual acuity outcomes PO: Postoperative

Poor vision	Impaired vision	Good vision	Mean value	
15 (10.3%)	50 (34.2%)	81 (55.5%)	0.47 (20/60)	PO-day 1
8 (6.4%)	25 (20%)	92 (73.6%)	0.33 (20/40)	PO-week 1
9 (6.6%)	28 (20.4%)	100 (73%)	0.32 (20/40)	PO-month 1
3 (3.3%)	26 (28.3%)	63 (68.5%)	0.33 (20/40)	PO-month 3
4 (4.7%)	14 (16.3%)	68 (79.1%)	0.32 (20/40)	PO-month 6
8 (6.3%)	16 (12.7%)	102 (81%)	0.28 (20/40)	PO-year 1

The incidence of RD was significantly higher in eyes with intraoperative posterior capsular rupture, zonular dehiscence, corneal trauma, and surgery combined with anterior vitrectomy than in other eyes (Table 7). The odds ratios for developing retinal detachment in patients with intraoperative posterior capsular rupture and in those undergoing anterior vitrectomy were 14.3 (95% CI 3.3-63.3) and 5.3 (95% CI 1.3-20.9), respectively (Table 8).

**Table 7:** Differences between the RD and non-RD groups RD: Retinal detachment

Retinal detachment	
P=0.647	Sex
P=0.198	Affected eye
P=0.483	Fundus exam abnormality
P=0.531	Myopia
P=0.132	Diabetes mellitus
P=0.004	IO posterior capsular rupture
P=0.065	IO vitreous loss
P=0.031	IO zonular dehiscence
P=0.033	Corneal trauma
P=0.037	Combined with anterior vitrectomy
P=0.064	Duration of surgery
P=0.011	Postoperative vision 1^st^ day
P=0.084	Postoperative vision 1^st^ month
P=0.030	Postoperative vision 1^st^ year

**Table 8:** Odds ratios for the risk of RD RD: Retinal detachment; CI: Confidence interval

95% CI	Odds ratio	
0.5–5	1.5	Male sex
0.7–10.4	2.8	Surgery in the right eye
0.2–11.7	1.4	Myopia
0.4–9.2	1.9	Glaucoma
0.8–17.3	3.7	Diabetes
3.3–63.3	14.3	Posterior capsular rupture
1.3–20.9	5.3	Anterior vitrectomy

The mean postoperative intraocular pressure was 15.5 mmHg, ranging from 5 to 50 mmHg.

The most commonly reported complication after cataract surgery was corneal edema in 22.7% of cases (n=83). Other complications are shown in Table 9. The prolonged surgical duration was significantly associated with the rate of corneal edema (P=0.043).

**Table 9:** Incidence of postoperative complications

No	Yes	
353 (96.7%)	12 (3.3%)	Retinal detachment
365 (100%)	0 (0%)	Endophthalmitis
358 (98.1%)	7 (1.9%)	Posterior capsular opacification
362 (99.2%)	3 (0.8%)	Anterior uveitis
363 (99.5%)	2 (0.5%)	Glaucoma
282 (77.3%)	83 (22.7%)	Corneal edema
364 (99.7%)	1 (0.3%)	Iris prolapse
361 (98.9%)	4 (1.1%)	Iris atrophy
362 (99.2%)	3 (0.8%)	Lens subluxation
362 (99.2%)	3 (0.8%)	Capsular phimosis

## Discussion

In the present study, we aimed to estimate the incidence and risk factors of RD following cataract surgery. We reported that 3.3% developed RD after the surgery, which is higher than the rate of 0.08 that was estimated for the general population [4]. A retrospective study conducted in France that examined data for 2,680,167 eyes that underwent cataract surgery over four years reported an overall RD incidence of 0.99% while another study involving 46 hospitals in Australia reported an overall RD incidence of 0.7% [5,6]. A retrospective study conducted in Korea reported a higher incidence of RD after cataract surgery (approximately 1.19%) although the rate was even higher in the study by Laube (3.55%); this was close to our observed incidence in the present cohort study [11,12].

Most patients with RD in our study were male, as the risk of RD is 1.5 times higher in men in compared to women. This finding is in accordance with established evidence for male sex as a risk factor for RD, which has been shown to increase risk by 2 to 2.5 folds [5,13,14]. In the current study, the risk of RD was 2.8 times higher in the right eye than in the left eye; this was in accordance with the results of a systemic review of 16 papers conducted by Qureshi [15].

In our analysis of risk factors for RD, posterior capsular rupture and anterior vitrectomy exerted the most significant effects on the risk of RD. Multiple studies have reported similar results. A 2003 study conducted among 324 patients with posterior capsule rupture in Sweden reported a 10-fold increase in the risk of RD while another study reported a four-fold increase in RD risk among those with posterior capsular rupture [6,16].

Another study conducted in the United Kingdom that included data for 18,065 eyes assessed the differences in RD risk for a posterior capsular rupture with or without vitreous loss. The authors reported that the RD risk was 13 to 16 times higher in eyes with posterior capsular rupture and vitreous loss than in eyes with an intact capsule. However, no cases of RD were observed among eyes with posterior capsular rupture and no vitreous loss [14]. Our analysis also revealed a significant association between intraoperative zonular dehiscence and RD, consistent with the results of another case­-control study by Tuft [17].

In our study, mean visual acuity improved from 20/80 preoperatively to 20/60 on postoperative Day 1 and to 20/40 thereafter. A study conducted at Moorfield Eye Hospital reported that visual acuity improved from 0.5 (20/60) to 0.2 (20/30), similar to our findings [18]. Another cross-sectional study assessed the difference in visual acuity between patients with and without diabetes, reporting that 87% of those with diabetes achieved normal vision (20/20 to 20/60) on postoperative Day 1, in contrast to 92% of those without diabetes. However, in our study, only 55.5% of patients achieved normal vision on postoperative Day 1 [19].

The most common complication of cataract surgery was corneal edema, which was observed in 22.7% of the eyes, and our analysis revealed a significant relationship between corneal edema and the duration of surgery (P-value = 0.043). Previous research has reported a lower risk of corneal edema after femtosecond laser-assisted cataract surgery when compared with that after phacoemulsification secondary, which may be related to differences in surgical duration [20].

This study had some limitations, as some variables of interest such as axial length were unavailable. Additionally, some patient data were missing from medical records, and loss to regular follow-up occurred in some cases. Lastly, we were unable to analyze the difference in the risk of RD between phacoemulsification and extracapsular cataract extraction, as all patients in the current study underwent phacoemulsification.

## Conclusions

In the current study, the rate of RD following cataract surgery was higher than that reported in most previous studies at one month postoperatively. This might be due to non-compliance with regular follow-up in some patients; another explanation is that we didn't take all eyes operated on during the studied period.

In addition, our analysis identified male sex, diabetes mellitus, posterior capsular rupture, and anterior vitrectomy as risk factors for RD. We also observed that RD was more common in the right eye than in the left eye. Significant correlations were further observed between RD and posterior capsular rupture, anterior vitrectomy, and zonular dehiscence.
